# ILB^®^ resolves inflammatory scarring and promotes functional tissue repair

**DOI:** 10.1038/s41536-020-00110-2

**Published:** 2021-01-07

**Authors:** Lisa J. Hill, Hannah F. Botfield, Ghazala Begum, Omar Qureshi, Vasanthy Vigneswara, Imran Masood, Nicholas M. Barnes, Lars Bruce, Ann Logan

**Affiliations:** 1 School of Biomedical Sciences, Institute of Clinical Sciences, University of Birmingham, Birmingham, B15 2TT UK; 2 Neuroscience and Ophthalmology, Institute of Inflammation and Ageing, University of Birmingham, Birmingham, B15 2TT UK; 3 School of Pharmacy, Institute of Clinical Sciences, University of Birmingham, Birmingham, B15 2TT UK; 4 TikoMed AB, P.O. Box 81, 263 03 Viken, Sweden; 5 Axolotl Consulting Ltd., Droitwich, Worcestershire WR9 0JS UK; 6 Biomedical Sciences, Warwick Medical School, University of Warwick, Coventry, CV4 7AL UK

**Keywords:** Mechanisms of disease, Vision disorders, Drug development

## Abstract

Fibrotic disease is a major cause of mortality worldwide, with fibrosis arising from prolonged inflammation and aberrant extracellular matrix dynamics. Compromised cellular and tissue repair processes following injury, infection, metabolic dysfunction, autoimmune conditions and vascular diseases leave tissues susceptible to unresolved inflammation, fibrogenesis, loss of function and scarring. There has been limited clinical success with therapies for inflammatory and fibrotic diseases such that there remains a large unmet therapeutic need to restore normal tissue homoeostasis without detrimental side effects. We investigated the effects of a newly formulated low molecular weight dextran sulfate (LMW-DS), termed ILB^®^, to resolve inflammation and activate matrix remodelling in rodent and human disease models. We demonstrated modulation of the expression of multiple pro-inflammatory cytokines and chemokines in vitro together with scar resolution and improved matrix remodelling in vivo. Of particular relevance, we demonstrated that ILB^®^ acts, in part, by downregulating transforming growth factor (TGF)β signalling genes and by altering gene expression relating to extracellular matrix dynamics, leading to tissue remodelling, reduced fibrosis and functional tissue regeneration. These observations indicate the potential of ILB^®^ to alleviate fibrotic diseases.

## Introduction

Fibroproliferative diseases are a major cause of mortality and morbidity worldwide, with an estimated 45% of deaths being related to fibrotic diseases. Aberrant fibrosis subsequent to prolonged or unresolved inflammation occurs throughout the body, and contributes to most deaths associated with common age-related systemic and vascular diseases, including neural, cardiac, hepatic and pulmonary fibrosis^1^. Personal and societal burdens of inflammation and fibrosis also occur in non-life threatening, but severely debilitating diseases, including blindness from glaucoma, estimated to affect 76 million people worldwide^2^. In primary open angle glaucoma (POAG; the most prevalent form), excessive extracellular matrix (ECM) deposition within the trabecular meshwork (TM) results in increased resistance to outflow of aqueous humour (AqH) causing ocular hypertension and consequent damage to retinal neurons leading to neurodegeneration and irreversible blindness^3^.

Despite improved knowledge linking inflammation, dysregulated ECM dynamics and fibrosis, there has been limited success in translating effective anti-fibrotic treatments from pre-clinical models. Targeting only inflammation (with anti-inflammatories) has not been successful in preventing the onset of fibrosis and more recent anti-fibrotic therapies have shown limited success in clinical efficacy^1^. Therefore, efficacious therapies that resolve inflammation, arrest aberrant pro-fibrotic pathways and activate functional tissue regeneration would be of significant societal and economic value to effectively treat many of these devastating diseases.

The exact inflammatory mechanisms that lead to pathogenic fibrosis are disease and tissue dependent, but there are common pathways that present an opportunity for drug development. Multi-modal drugs that can target multiple signalling pathways have the potential to not only resolve inflammation but simultaneously regulate ECM dynamics to reduce fibrosis. The pro-fibrotic cytokine, transforming growth factor β (TGFβ), has been extensively studied in this context and orchestrates inflammation and fibrosis through recruitment, activation and proliferation of innate and adaptive immune cells, epithelial cells and fibroblasts, whilst regulating ECM dynamics. Pre-clinical studies suggest inhibition of TGFβ displays considerable therapeutic potential, yet translation to the clinic has been challenging^1^. This perhaps highlights the limitations inherent in targeting single inflammatory/fibrotic pathways (e.g. using TGFβ-specific antibodies). Against this background it is of interest that agents targeting multiple inflammatory pathways, e.g. decorin, have progressed successfully through pre-clinical models^4–6^ and now head towards first in man trials for ocular scarring diseases. Clinically, the most notable anti-fibrotic treatments, pirfenidone and nintedanib, have recently received marketing authorisation by the FDA with an indication for lung fibrosis^7^. Pirfenidone and nintedanib exert their anti-fibrotic effects via suppression of TGFβ and tumour necrosis factor α (TNFα) synthesis, however, only modest improvements in lung function and survival have been reported^8^, highlighting the continued need for better anti-fibrotic medication.

Another class of molecules, dextran sulfates, display favourable modulation of wound healing processes and have been assessed in clinical trials as anticoagulant agents^9,10^. Conversely, oral administration of dextran sulfate sodium has been used experimentally to induce inflammatory diseases in animal models (e.g. inflammatory bowel disease^11^). These apparent opposing anti- or pro-inflammatory effects are attributed to their different structures and molecular size, with the larger dextran sulfate sodium being more pro-inflammatory^12^ and low molecular weight formulations showing favourable anti-inflammatory and anti-fibrotic actions^13^. Recently low molecular weight dextran sulfates (LMW-DS) have shown efficacy in rodent studies by reducing immune cell recruitment and tissue fibrosis in cardiac^14^ and diabetic^13,15^ experimental models. A novel LMW-DS formulation (International patent application no. WO 2016/076780^16^), ILB^®^, evokes unique biological effects and displays low toxicity compared to other investigated dextran sulfates. Already assessed in human trials, ILB^®^ displays an acceptable safety profile in healthy volunteers, with relatively weak anticoagulant effects^17^.

In this study we sought to understand the mechanism and effects of ILB^®^ on inflammatory mediators and wound repair in cultured primary human cells using the BioMap® phenotypic profiling platform^18^, gene expression profiling, as well as evaluating the drug’s ability to modulate ECM remodelling in fibroproliferative conditions in vivo, using a disease-relevant rodent model of anterior segment fibrosis to mimic the fibroproliferative pathologies evident in some patients with POAG.

## Results

### ILB^®^ modulates inflammatory and wound healing responses in cultured human cells

**Fig. 1:**
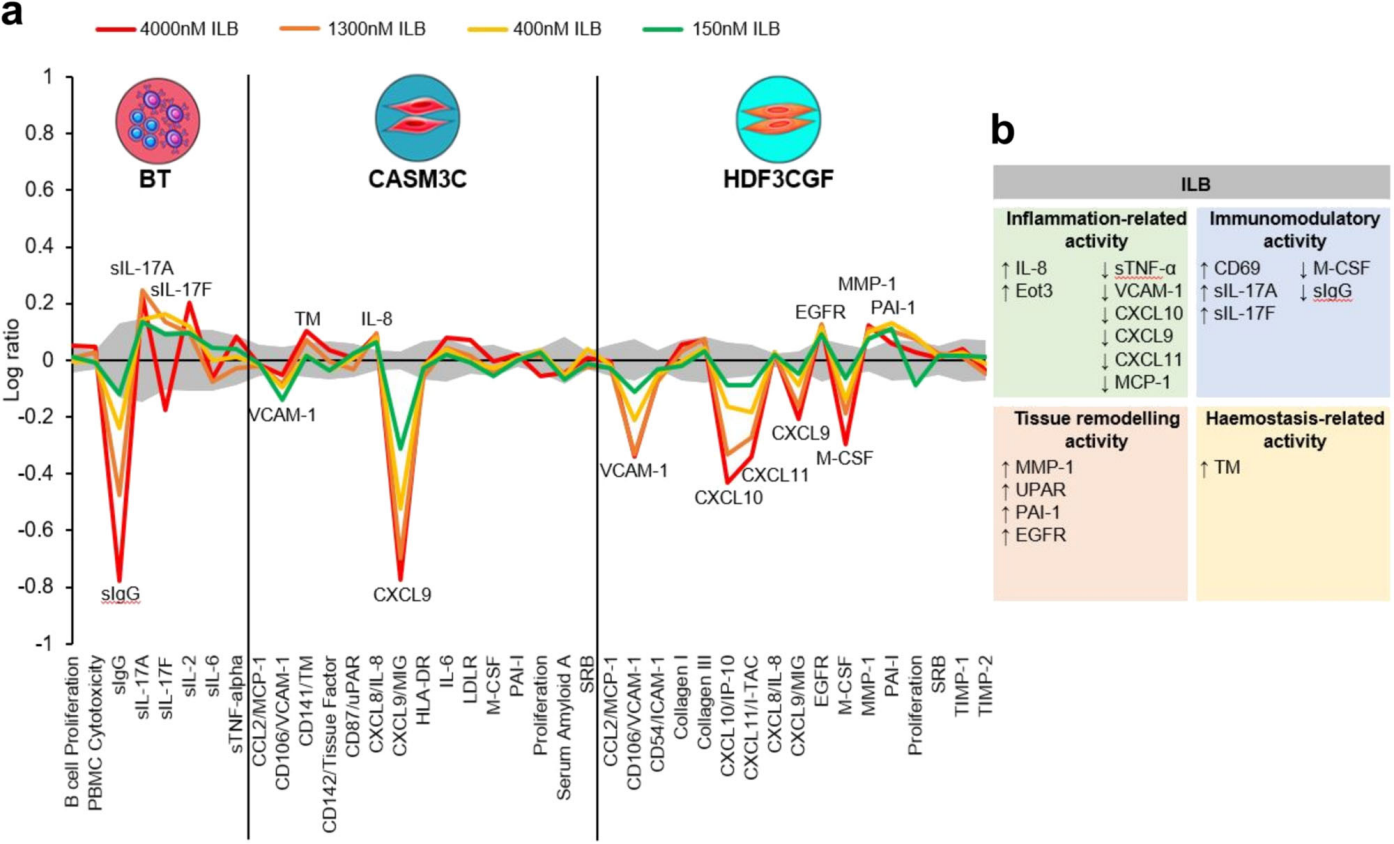
BioMAP^®^ Diversity Plus activity profiles for ILB^®^. (**a**) Log ratio data relating to the cellular and molecular responses of human BT (B cells+peripheral blood mononuclear cells), CASM3C (coronary artery smooth muscle cells) and HDF3CGF (dermal fibroblasts) systems to 150 (green line), 400 (yellow line), 1300 (orange line) and 4000 (red line) nM of ILB^®^. The biomarkers assessed in each system are indicated along the *x*-axis and the grey envelope represents historical vehicle control data at a 95% confidence interval. Biomarkers that are deemed to be significantly altered by ILB^®^ treatment are annotated on the graph (n=minimum of 6). (**b**) Summary of significant ILB^®^-induced biomarker changes across all 12 BioMAP^®^ Diversity Plus human cell systems. The changed biomarkers fall into 4 biological activities that are particularly relevant to the observed anti-inflammatory and fibrolytic effects of ILB^®^ and indicate how ILB^®^ modulated their activity.

BioMAP^®^ has been developed as a method to assess efficacy, safety and the mechanism of action of drugs in multiple human cell types stimulated with inflammatory challenges. Of the twelve BioMAP^®^ human cell culture systems selected to model inflammation and wound healing, three cell systems demonstrated three or more unique changes in cellular responses with ILB^®^ treatment: the BT, CASM3C and HDF3CGF systems. For example, ILB^®^ reduced the production of soluble immunoglobulin G (sIgG) but increased the secretion of interleukin (IL) -17A and IL-17F (Fig. 1a) in the BT system, which relates to T cell-dependent activation of B cells. In the CASM3C system, which represents the Th1 inflammatory environment for arterial smooth cells, ILB^®^ reduced vascular cell adhesion molecule 1 (VCAM-1) and C-X-C motif chemokine ligand 9 (CXCL9) while increasing the levels of thrombomodulin (TM) and IL-8 (Fig. 1a). The greatest response to ILB^®^ treatment was observed in the tissue remodelling responses of human dermal fibroblasts (HDF3CGF system), with ILB^®^ reducing the levels of VCAM-1, CXCL9, CXCL10, CXCL11 and macrophage colony-stimulating factor (M-CSF), and increasing the amount of epidermal growth factor receptor (EGFR), matrix metalloproteinase-1 (MMP-1) and plasminogen activator inhibitor-1 (PAI-1; Fig. 1a). Importantly, there was no cytotoxicity evident with ILB^®^ in any of the human culture systems evaluated. Overall analysis of the responses of the human cell model systems included in the assay showed that ILB^®^ attenuated inflammatory and immunoregulatory cytokines and molecules, whilst enhancing tissue remodelling pathways (including activation of matrix degrading enzymes) and haemostasis activity (Fig. 1b). We benchmarked these effects against that of an approved anti-fibrotic drug, pirfenidone, and demonstrated that ILB^®^ induced a unique anti-inflammatory profile, by targeting different inflammatory regulators after challenge in this broad range of human cell systems (Supplementary Fig. 1).

### ILB^®^ modulates expression of inflammatory and fibrogenic genes in cultured human cells

**Fig. 2:**
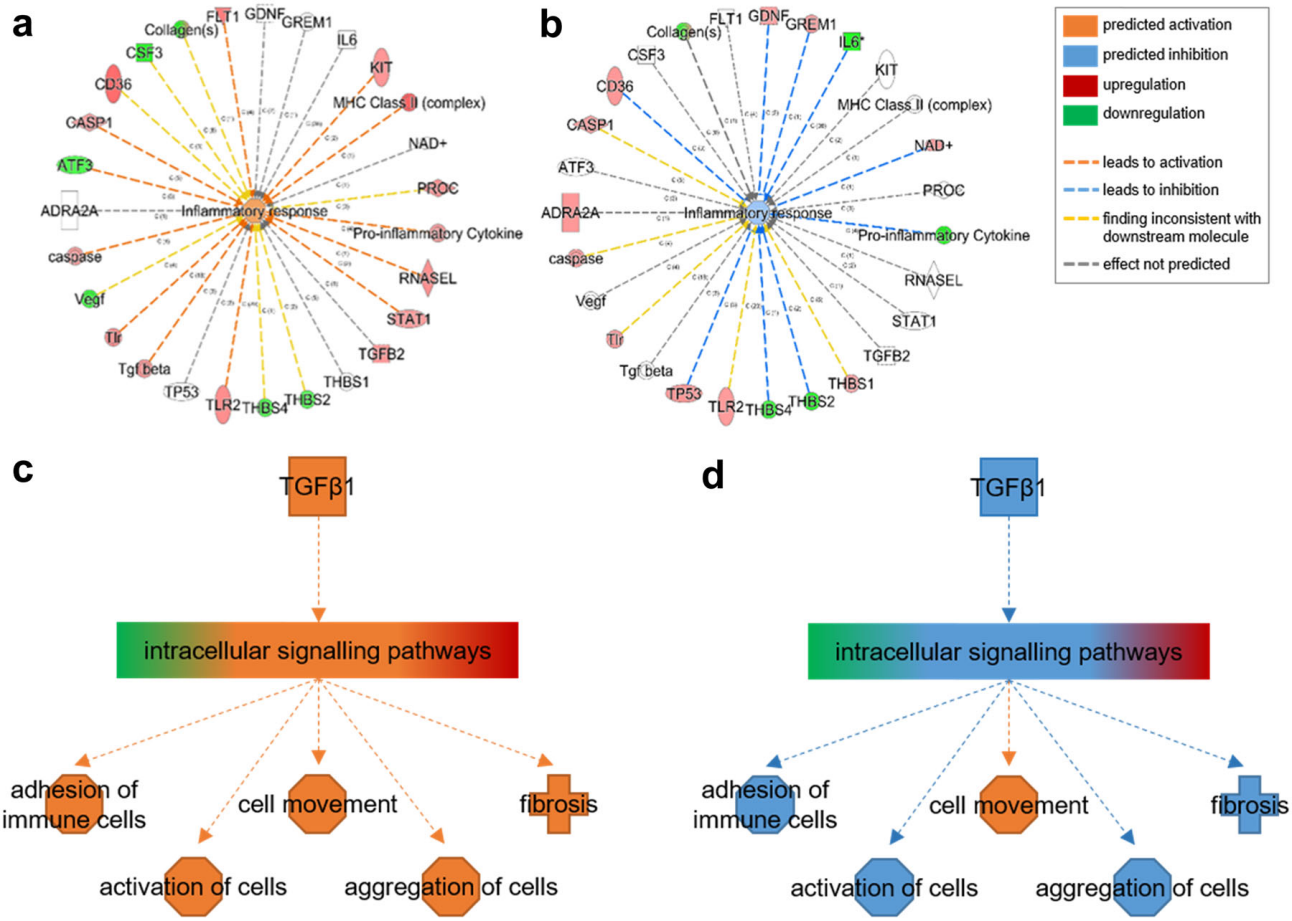
ILB^®^ modulation of inflammation and TGFβ1 pathways. Summary of the TGFβ signalling genes that were differentially regulated in human Schwann cell cultures over 48 h in the absence (**a**, **c**) and presence of ILB^®^ (**b**, **d**) (*n* = 3). When placed in culture the Schwann cells exhibit a pro-inflammatory phenotype, i.e. responding to endogenous pro-inflammatory cytokines, including TGFβ, that initiate a cascade of inflammatory responses (**a**, **b**). Red denotes the genes upregulated in the Agilent gene array data set, green indicates the genes downregulated in the Agilent array data set. TGFβ signalling (a pro-inflammatory cytokine) regulates the expression of a large number of molecules that control numerous cellular responses. The known downstream molecular interactions of TGFβ signalling molecules (derived from the IPA knowledge base) allow the prediction of the downstream cellular effects of TGFβ modulation (**c**, **d**). The orange inflammatory designation indicates the activation of an inflammatory cellular response to the pro-inflammatory gene expression profile seen under normal culture conditions (**c**); the blue anti-inflammatory designation shows the resolution of inflammation seen after inhibition of the expression of pro-inflammatory genes by ILB^®^ (**d**). In the human Schwann cell cultures (**c**), TGFβ activation elicits the activation of immune cells and fibrosis and while ILB^®^ treatment (**d**) inhibits these changes, with the exception of cell movement.

To confirm and further understand the mechanisms of action of ILB^®^, and to underline the broad relevance of ILB^®^ actions, we analysed the drug impact upon the expression of genes of particular relevance to inflammation and tissue repair in human Schwann cells; these glia are widely used to evaluate inflammatory and fibrotic responses seen in many neurodegenerative disorders. The functional analysis of the differentially regulated genes relevant to inflammatory scarring in the Schwann cell cultures (baseline and ILB^®^ treated) indicates that, while the TGFβ signalling was not significantly affected in the cultures at baseline, the pathway was significantly affected by ILB^®^. All subsequent analyses were focused on pathways and molecules relevant to POAG and the animal models used in this study. Three different analyses were carried out on the gene expression data; the first was focussed on assessing ILB^®^ modulation of immune response genes (Fig. 2a, b), the second focussed on genes involved in the TGFβ signalling pathways (Fig. 2c, d, Supplementary Fig. 2 and Supplementary Table 1) and the third was looking more specifically at the links between TGFβ and fibrosis (Supplementary Table 2).

Gene expression analysis demonstrated that, when placed in culture, human Schwann cells produce a robust inflammatory gene expression profile resulting in activation of an inflammatory response (Fig. 2a) that is very similar to that activated by TGFβ (Supplementary Table 2). Similar to the BioMAP^®^ proteomic data, molecular network analysis showed that ILB^®^ had a complex effect on inflammatory genes, resulting in resolution of the normal inflammatory response, in part by preventing the upregulation of genes such as TGFβ, MHC class II complex and ribonuclease L (RNASEL), and downregulating genes for pro-inflammatory cytokines and IL-6 (Fig. 2b). We further assessed the influence of ILB^®^ on modulating key elements of the signalling cascade relating to the pro-fibrotic cytokine TGFβ.

Whilst response-activated TGFβ gene expression was attenuated by ILB^®^, diverse elements of the downstream TGFβ signalling cascade were also influenced. In Schwann cells ILB^®^ was predicted to inhibit pathways involved in tissue remodelling, including protease activation, cell migration, adhesion of immune cells, activation of cells, aggregation of cells and fibrosis (Fig. 2c, d, full gene network shown in Supplementary Fig. 2 and Supplementary Table 1). Some of these tissue remodelling genes are common to fibrogenic as well as fibrolytic cellular responses, for example, protease activity regulators like ADAM/SERPIN/A2M and cell adhesion/migration molecules like V-CAM/PSTN. The TGFβ-regulated mechanistic molecular network responsible for fibrosis includes 165 molecules and, of these, ILB^®^ significantly regulated the expression of 14 (8.5%; *p* < 0.001). The changes in gene expression in this TGFβ-regulated gene network indicate that ILB^®^ attenuated fibrosis in part by upregulating the expression of the anti-fibrotic proteoglycan decorin (DCN; Fold Change = 1.242), cell adhesion/migration proteins like V-CAM/periostin (POSTN; Fold Change = 1.212) and also matrix metalloproteases and their inhibitors (ADAM17 Fold Change = 1.238; ADAMTS9 Fold Change = 1.24; A2M Fold Change = 1.271; SERPIN family members: SERPINB2 Fold Change = 1.283, SERPINB7 Fold Change = 1.352, SERPINE2 Fold Change = 1.204), thereby activating tissue remodelling processes (Supplementary Table 2). It is also noteworthy that, while not all elements of the TGFβ response are affected by ILB^®^, the overall downstream effect of the drug is modulation of the TGFβ signalling pathway. The comparison data also show that the effects of ILB^®^ are significantly different from heparin despite the structural similarity between the two.

### ILB^®^ reduces fibronectin levels in cultured human trabecular meshwork cells and resolves inflammatory scarring in a rodent model of glaucoma

**Fig. 3:**
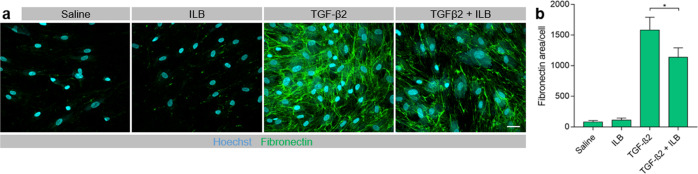
ILB^®^ modulation of the molecular pathways involved in fibrosis. Human trabecular meshwork cells were stimulated with TGFβ2 (1.0 ng/ml) in the absence (saline) or presence of ILB^®^ (4.0 µM) for 72 h followed by immunofluorescence labelling of fibronectin (green) and nuclei staining with Hoechst (cyan). (**a**) Representative images are maximum intensity projections from confocal Z-stacks (scale bar = 40 µm) and (**b**) the histogram shows quantification of the fibronectin staining normalised to the number of cells (mean ± SEM, *n* = 6), *P* < 0.05 (Wilcoxon test).

**Fig. 4:**
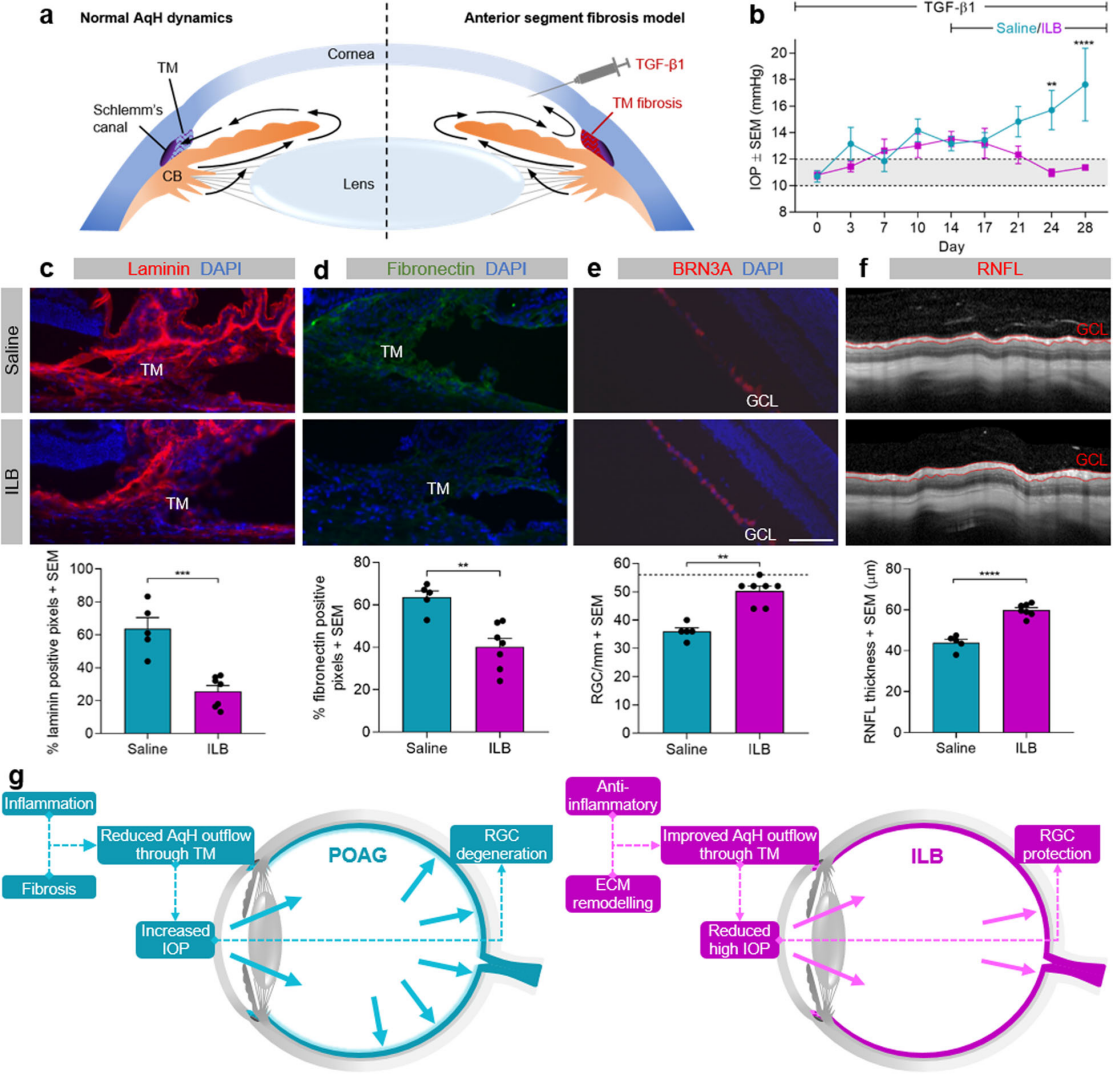
Subcutaneous injections of ILB^®^ reduces IOP and TM ECM molecules and increases RGC survival in a rodent model of anterior segment fibrosis. (**a**) Schematic diagram showing the method for inducing the anterior segment fibrosis model. Twice weekly intracameral injections of TGFβ1 induced fibrosis in the trabecular meshwork (TM), blocking AqH outflow and raising IOP. (**b**) Line graph showing IOP measurements during IC TGFβ1 treatment for the first 14 days followed by IC TGFβ1 with daily subcutaneous saline vehicle control or ILB^®^ treatment for a further 14 days. Normal IOP levels are indicated by the grey shaded area, **P < 0.01, ****P < 0.0001 (2-way ANOVA). (**c**, **d**) Representative images of ocular tissue sections including the angle of the anterior segment together with related histograms showing levels of immunoreactive laminin (red) and fibronectin (green) staining in the TM in the saline and ILB^®^ treatment groups, **P < 0.01 ***P < 0.001 (t-test). (**e**) Representative images and histogram showing the RGC marker BRN3a (red, arrows) in retinal sections from the saline and ILB^®^ treatment groups, **P < 0.01 (Mann–Whitney test), GCL—ganglion cell layer. Scale bar = 100 µm. (**f**) Representative optical coherence tomography images and related histogram showing the segmented RNFL (red outline) in the saline and ILB^®^ treatment groups, ****P < 0.0001 (t-test). Saline group *n* = 5, ILB^®^ group *n* = 7. (**g**) Schematic diagram showing the potential mechanism of ILB^®^ in POAG. The IOP data are described in the ILB^®^ patent^33^.

POAG is an example of a chronic ocular fibrotic disease. In the eye, TGFβ is known to increase the deposition of ECM in the TM, most notably fibronectin^19,20^. Fibronectin is found within the sheath material linking the innermost region of the TM with Schlemm’s canal and helps to regulate the tissue’s contractile properties and consequently AqH drainage and intraocular pressure (IOP)^20^. Higher levels of TGFβ and fibronectin have been detected in the AqH of patients with glaucoma compared to controls^20,21^. Here we demonstrated that ILB^®^ significantly reduced fibronectin levels in cultured human trabecular meshwork cells treated with TGFβ2 (1136 ± 155 *vs* 1579 ± 210; *P* < 0.05; Fig. 3a, b). Following this, we assessed the effects of subcutaneous (SC) injections of ILB^®^ in a rodent model of anterior segment fibrosis, an experimental model of glaucoma, in which TM fibrosis is induced through bi-weekly intracameral (IC) injections of TGFβ1^5^ (Fig. 4a). Twice weekly IC TGF-β1 injections significantly raised IOP to 13 mmHg by day 14 by establishing TM fibrosis, at which point daily SC injections of ILB^®^ or saline commenced. The IOP continued to rise in control rats receiving IC TGFβ1 plus SC injections of saline, reaching 17.6 ± 2.7 mmHg on day 28 (Fig. 4b). By contrast, IC TGFβ1 with SC ILB^®^ injections reduced IOP to 11.4 ± 0.2 mmHg by day 28 (*P* < 0.0001; Fig. 4b), which was within the normal range (10–12 mm Hg). Immunohistochemical analysis of fibrosis in the TM showed significant reductions in the ECM molecules laminin (25.6 ± 3.5%, *P* < 0.001; Fig. 4c) and fibronectin (40.2 ± 4.0%, *P* < 0.01, Fig. 4d) compared to the TM in SC saline-treated control rats (63.7 ± 6.8% and 63.6 ± 2.9%, respectively). This was accompanied by significantly enhanced numbers of surviving retinal ganglion cells (RGC) (12.6 ± 0.4 RGC/mm in SC ILB^®^-injected rats compared to 9.0 ± 0.3 RGC/mm in control SC saline-injected rats; *P* < 0.01; Fig. 4e) and preserved retinal nerve fibre layer (RNFL) thickness (59.9 ± 1.2 µm in SC ILB^®^-injected rats compared to 44.0 ± 1.6 µm in control SC saline-injected rats; P < 0.01; Fig. 4f) as measured by optical coherence tomography (OCT).

## Discussion

As inflammation precedes fibrosis in fibroproliferative conditions, it was important to understand how ILB^®^ might modulate inflammatory and tissue remodelling pathways in human cells. Protein and gene expression data from relevant cultured human cells suggest that, through reprogramming immune activation, inflammation resolution and tissue remodelling, ILB^®^ modulates key innate and adaptive responses leading to improved healing and functional remodelling of diseased and damaged human tissues.

We explored the mechanistic profile of ILB^®^ at the protein level using the BioMAP^®^ Diversity Plus assay, which comprised 12 different validated human primary cell culture systems that, when challenged, modelled inflammation and wound healing responses in different human tissue types. The data demonstrated that ILB^®^ had a distinct phenotypic profile with multi-modal actions; having specific effects in different cell types following stimulation with different inflammatory mediators. In particular, ILB^®^ reduced the levels of 3 chemokines, CXCL9, CXCL10 and CXCL11, which are all structurally related, signal through the CXC receptor 3 (CXCR3) and are induced by interferons and TNFα. CXCR3 and its associated chemokines play an important role in the recruitment and function of immune cells and have been implicated in numerous inflammatory and autoimmunity diseases^22,23^. In addition, they have been used as prognostic and predictive biomarkers of disease. The CXCR3 pathway plays an important role in the aetiology of glaucoma, with expression levels correlating with the progression of POAG^24^. In models of ocular hypertension, antagonism of CXCR3 reduces IOP, increases AqH outflow, reduces inflammation and reduces apoptosis of TM and retinal cells^25,26^. Therefore, this would be an interesting mechanistic area to investigate further for ILB^®^.

Other dextran derivatives have been shown to reduce ECM deposition and TGFβ expression, in addition to increasing anti-fibrotic proteoglycans and macrophage infiltration in fibrotic models^27^. However, both the gene expression data and the results from the glaucoma model reported here support a unique multi-modal influence of ILB^®^, due to its actions on the TGFβ signalling pathway as well as on other ECM remodelling signals. In POAG, TGFβ has a role in the progressive accumulation of ECM proteins in the TM, preventing normal outflow of AqH leading to elevated IOP. The rodent model of glaucoma used in this study models TGFβ induced anterior segment fibrosis and provides a robust, clinically relevant means of evaluating anti-fibrotic therapies. Here we demonstrated that daily SC injections of ILB^®^ resolved inflammatory signalling, reversed established TM fibrosis and lowered IOP, thereby rescuing RGC from progressive death, supporting the potential for ILB^®^ to modify the progression of fibroproliferative disorders by modulating the TGFβ pathway. Furthermore, these results achieved with daily SC injections of ILB^®^ are comparable to those evident in the same in vivo model with twice weekly IC injections of decorin, which displays similar anti-fibrotic efficacy in experimental models of eye^5,6,28^, spinal cord^29^ and brain fibrosis^30,31^. Interestingly, ILB^®^ may act in part to modulate inflammation and promote tissue remodelling by increasing decorin expression, as shown in the gene expression analysis. A potential upstream mechanism of action of ILB^®^ could be by mimicking some aspects of heparin activity^32^ and directly binding TGFβ (along with other heparin-binding cytokines), thereby preventing TGFβ from binding to its receptor thus blocking subsequent signalling cascades and downstream inflammatory/fibrotic cellular responses. However, it should be emphasised that the pattern of gene expression responses seen to be elicited by ILB^®^ is distinct to that of heparin and other scar regulators, thus differentiating the mechanism of action of ILB^®^.

In conclusion, this is the first study to demonstrate that SC injections of ILB^®^, which has already demonstrated safety in humans, can resolve inflammation and nascent/established fibrosis in both rodent and human models of inflammation and fibrosis, thereby supporting functional tissue regeneration. As a consequence, in the ocular disease model evaluated, inflammatory fibrosis was resolved, IOP was rapidly normalised and compromised retinal cells were protected from progressive death. We have also provided insights into the multi-modal mechanism of action of ILB^®^. Taken together, our studies provide proof of concept that ILB^®^ has potential as a novel disease-modifying therapy for the treatment of POAG and other acute and chronic fibroproliferative conditions.

## Methods

### ILB^®^

ILB^®^ is a unique and distinct LMW-DS formulation whose structure, formulation, synthesis and relevance to fibrotic diseases like glaucoma has been described in detail previously in two published patents (WO 2016/076780^16^ and WO 2018/212708^33^). ILB^®^ (batch number 3045586) was provided by Tikomed AB, Viken, Sweden at a stock concentration of 20 mg/ml and was kept in a temperature monitored refrigerator. Immediately prior to use ILB^®^ aliquots were diluted to the appropriate concentration in sterile saline and used in cell culture or administered by subcutaneous injection in vivo.

### Phenotypic profiling using BioMAP^®^ Systems

Twelve primary human cell and co-culture assays were used to assess the effects of ILB^®^ on clinically relevant protein biomarkers of inflammation, cell growth, and fibrosis as part of the quality controlled BioMAP^*®*^ Diversity PLUS, commercially available service (Eurofins DiscoverX Corporation, Freemont, CA, USA; for full details https://www.discoverx.com). Briefly, the BioMAP^*®*^ panels consist of human primary cell-based systems designed to model different aspects of the human body in an in vitro format. The 12 cell assays utilised in the Diversity PLUS panel allow characterisation of test agent responses in an unbiased way across a broad set of systems modelling various human disease states compared to historical controls. BioMAP^*®*^ panels are constructed with primary cell types from healthy human donors, with stimuli (such as cytokines or growth factors) added to capture relevant signalling networks that naturally occur in human tissue or pathological conditions.

ILB^®^ was tested in these assays at four concentrations: 4000 nM, 1300 nM, 400 nM & 150 nM. Human primary cells employed in the BioMAP^*®*^ systems were used at passage 4 or earlier, derived from multiple donors (*n* = 2–6), commercially purchased and handled according to the recommendations of the manufacturers. Human blood derived CD14 + monocytes were differentiated into macrophages in vitro before being added to the lMphg system (Eurofins DiscoverX Corporation).

The human cell types and stimuli used in each assay system were as follows: 3 C system [human umbilical vein endothelial cells (HUVEC) + (IL-1β, TNFα and IFNγ)], 4H system [HUVEC + (IL-4 and histamine)], lipopolysaccharide (LPS) system [peripheral blood monocyte cells (PBMC) and HUVEC + LPS (TLR4 ligand)], SAg system [peripheral blood mononuclear cells, PBMC and HUVEC + TCR ligands], BT system [CD19 + B cells and PBMC + (α-IgM and TCR ligands)], BF4T system [bronchial epithelial cells and human neonatal dermal fibroblasts, HDFn, + (TNFα and IL-4)], BE3C system [bronchial epithelial cells + (IL-1β, TNFα and IFNγ)], CASM3C system [coronary artery smooth muscle cells + (IL-1β, TNFα and IFNγ)], HDF3CGF system [HDFn + (IL-1β, TNFα, IFNγ, EGF, bFGF and PDGF-BB)], KF3CT system [keratinocytes and HDFn + (IL-1β, TNFα, IFNγ and TGFβ)], MyoF system [differentiated lung myofibroblasts + (TNFα and TGFβ)] and lMphg system [HUVEC and M1 macrophages + Zymosan (TLR2 ligand)].

Assays were derived from either single cell types or co-culture systems. Adherent cell types were cultured in 96 or 384-well plates until confluence, followed by the addition of PBMC (SAg and LPS systems). The BT system consisted of CD19 + B cells co-cultured with PBMC and stimulated with a BCR activator and low levels of TCR stimulation. Test agents prepared in either DMSO (small molecules; final concentration ≤ 0.1%) or PBS (biologics) were added at the indicated concentrations 1 h before stimulation, and cells remained in culture for 24 h or as otherwise indicated [48 h, MyoF system; 72 h, BT system (soluble readouts); 168 h, BT system (secreted IgG)]. Each assay plate contained drug controls (e.g., legacy control test agent colchicine at 1.1 μM), negative controls (e.g., non-stimulated conditions) and vehicle controls (e.g., 0.1% DMSO) appropriate for each system. Direct ELISA was used to measure biomarker levels of cell-associated and cell membrane targets. Soluble factors from supernatants were quantified using either HTRF^®^ detection, bead-based multiplex immunoassay, or capture ELISA. Overt adverse effects of ILB^®^ on cell proliferation and viability (cytotoxicity) were detected by sulforhodamine B (SRB) staining for adherent cells, and alamarBlue^®^ reduction for cells in suspension. For proliferation assays, individual cell types were cultured at subconfluence and measured at time points optimised for each system (48 h: 3 C and CASM3C systems; 72 h: BT and HDF3CGF systems; 96 h: SAg system). Cytotoxicity for adherent cells was measured by SRB (24 h: 3 C, 4H, LPS, SAg, BF4T, BE3C, CASM3C, HDF3CGF, KF3CT, and lMphg systems; 48 h: MyoF system), and by alamarBlue^®^ staining for cells in suspension (24 h: SAg system; 42 h: BT system) at the time points indicated.

### BioMAP^®^ data analysis

Biomarker measurements in ILB^®^-treated samples were divided by the average of the control samples (at least 6 vehicle controls from the same plate) to generate a ratio that was then log_10_ transformed. Significance prediction envelopes were calculated using proprietary historical vehicle control data at a 95% confidence interval. Biomarker activities were annotated when two or more consecutive concentrations change in the same direction relative to vehicle controls were outside of the significance envelope and had at least one concentration with an effect size > 20% (log_10_ ratio> 0.1). Biomarker key activities were described as modulated if these activities increase in some systems but decrease in others. Cytotoxic conditions were noted when total protein levels decreased by more than 50% (log_10_ ratio of SRB or alamarBlue^®^ levels < −0.3) and were indicated by a thin black arrow above the *X*-axis. A compound was considered to have broad cytotoxicity when cytotoxicity was detected in 3 or more systems. Concentrations of test agents with detectable broad cytotoxicity were excluded from biomarker activity annotation and downstream benchmarking, similarity search and cluster analysis. Antiproliferative effects were defined by an SRB or alamarBlue^®^ log_10_ ratio value < −0.1 from cells plated at a lower density and were indicated by grey arrows above the X-axis. Cytotoxicity and antiproliferative arrows only require one concentration to meet the indicated threshold for profile annotation.

### Gene expression studies

Human Schwann cells (sNF96.2, ATCC CRL-2884™, ATCC, Teddington, UK) were cultured in 25 cm^2^ culture flasks coated with poly-d-lysine and laminin) in high-glucose DMEM with 10% foetal bovine serum (FBS) (*n* = 8) and incubated at 37 °C/5%CO_2_. Twenty-four hours after seeding (Day 0) cells were treated once with either ILB^®^ (0.01 mg/ml in DMEM) (*n* = 3) or culture media (*n* = 3). The remaining 2 flasks of Schwann cells were used as the baseline cultures. After 48 h with treatment, the media were removed and cells were harvested in 2.5 ml Trizol:Water (4:1) solution containing 0.5 ml of chloroform at room temperature (RT). The flasks were inspected under the microscope to ensure full removal of cells. The collected lysates were stored at −80 °C.

### RNA extraction for gene expression studies

Lysates were left to equilibrate for 5 min at room temperature to permit the complete dissociation of nucleoprotein complexes. 1 ml lysate was removed from each sample and 200 µL of chloroform was added to each and the tube was shaken vigorously. Samples were stored at room temperature for 2–3 min and subsequently centrifuged at 12,000 *g* for 15 min at 4 °C. The mixture separated into three layers: a lower red phenol-chloroform phase, an interphase and a colourless upper aqueous phase. The top ¾ of the aqueous phase (containing the RNA) was transferred to a new clean Eppendorf tube. The RNA was precipitated from the aqueous phase by adding an equal amount of 100% ethanol. The precipitated RNA was fixed onto a Spin Cartridge, washed twice and dried. The RNA was eluted in 50 μL warm RNase-Free Water. The amount and quality of the purified RNA was measured by Nanodrop. The RNA was stored at −80 °C before transfer to Source Bioscience for Gene Array analysis (Source Bioscience, Nottingham, UK; for full details https://www.sourcebioscience.com). The additional QC from the array service provider (Source Bioscience) indicated that the RNA was high quality (no degradation) and the amounts were well within the parameters of the Low Input RNA Microarray from Agilent.

### Analysis of gene expression data

The background-corrected expression data from each sample were downloaded into a separate file. The background-corrected signal was log_2_ transformed for all samples for statistical analysis. To reduce the false discovery rate in the samples, the signals that were below ‘expression level’ were removed. The ‘below expression’ level was set at 5 for the log_2_ transformed expression values. The expression value of the MAPT gene, which is not normally expressed in Schwann cells, was <5. However, the expression of a ‘control’ housekeeping gene ACTB (A_32_P137939; ACTB; β-actin) was comfortably above 5.

### Statistical analysis of the gene expression data

We used MetaboAnalyst^34^ to analyse the gene expression profiles. Based on the expression pattern of the Control probes on each array it was decided to carry out Median Centring for all arrays before analysis to reduce the variability of the results. Additionally, a preliminary analysis was carried out to screen out genes that were not differentially expressed between any combinations of the three datasets. Simple, non-stringent ANOVA (p < 0.05) was carried out to look for patterns of expression. Probes with no changes across the three datasets were eliminated. The remaining probe sets were analysed for fold change and significance using Volcano plots (Qlucore, Lund, Sweden). A greater than ±20% change in the expression of a probe (FC ≥ 1.2 or FC ≤ 0.84 in the log2 data) means that the FC in gene expression was at least 2 and regarded as physiologically significant.

The comparison of Day 0 Control samples to the Day 2 Control samples allowed the analysis of the gene expression changes seen in the cells during normal culture conditions. The effect of ILB^®^ was regarded as the difference between the Day 2 Control versus the Day 2 ILB^®^ treated samples. The genes differentially regulated and the fold change values from this analysis were uploaded to the Ingenuity Pathway Analysis (IPA; Qiagen Ltd., Manchester, UK) software. Following a core analysis of the differential expression profiles, comparison analyses were performed to provide a complete profile of the ILB^®^ effects. The TGFβ regulated mechanistic molecular network of interest was built from genes known to be associated with fibrosis and scarring.

### Culture of TM cell populations

Human trabecular meshwork cells (TMC-6590; ScienCell Research Laboratories, Carlsbad, CA) were cultured in TMC (6591; ScienCell Research Laboratories) complete medium and maintained at 37 °C in 5% CO_2_ until reaching 80–90% confluency. Cells were plated at densities of 5000–10,000 cells per well in Greiner-Bio Cell 96 Well black Cell Culture Microplates (655090, Sigma) and left to adhere overnight. The following day cells were treated with TGFβ2 (1.0 ng/ml; Preprotech, London, UK) and/or ILB^®^ (4.0 μM; Tikomed AB) under serum-free conditions for 72 h (*n* = 6/treatment).

### Immunostaining and confocal imaging for TM cells

After 72 h treatment, cells were fixed using 4% PFA in PBS (28908, Thermo Fisher Scientific, Gloucester, UK) for 10 min, washed with PBS and then washed with PBS containing saponin-based permeabilization buffer (00-8333-56, Thermo Fisher Scientific). Cells were then immunostained with mouse anti-human Fibronectin Alexa Fluor^®^ 488 (1/200 dilution, 53-9869-82, Thermo Fisher Scientific) and nuclear stain Hoechst 33342 (H21492, Thermo Fisher Scientific) in permeabilizing buffer in the dark for 1 h at room temperature. Finally, cells were washed twice in PBS containing saponin-based permeabilization buffer and once in PBS prior to image analysis by confocal microscopy where images were acquired on a Yokogawa CQ1 spinning-disc microscope using a ×40 objective and appropriate excitation/emission settings for Hoechst and Alexa Fluor^®^ 488. *Z*-series were acquired and are displayed as maximum intensity *z*-projections. Images were analysed using the Yokogawa image analysis software.

### Experimental design for ocular hypertension model

Before induction of raised IOP, rats were randomly assigned into two groups (TGFβ1 plus saline vehicle and TGFβ1 plus ILB^®^ treatment). Induction of raised IOP was induced as described elsewhere^5^. Previously published studies with this in vivo experimental model included repeated intracameral (IC) injections of saline, which had no effect on IOP or inflammation^5^. Briefly, at 0 days, a 15° disposable blade was used to make a self-sealing tunnel through the cornea and this access point was employed for all subsequent twice weekly 3.5 µl IC injections of 5 ng/ml TGFβ1 (Peprotech, London, UK), carried out using sterile glass micropipettes (Harvard Apparatus, Kent, UK) over the 28-day experiment. IOP was also measured twice weekly (immediately prior to the IC injection) throughout the 28-day experiment. At 14 days, non-responders to TGFβ (i.e. their IOP did not increase above baseline at any time point) were excluded from further analysis to yield an inclusion of *n* = 7 rats (14 eyes) for ILB^®^ and *n* = 5 rats (10 eyes) for saline vehicle treatments. At 14 days, rats received either subcutaneous injections of 15 mg/kg ILB^®^ (Tikomed AB) or an equivalent saline volume, which continued daily for 14 days. OCT measurements of the retinal nerve fibre layer (RNFL) were taken at 28 days as a surrogate for RGC density and ocular tissues from both groups were processed for immunohistochemistry to assess levels of TM fibrosis and RGC survival.

### Animals and surgery

The rodent study was performed at the Biomedical Services Unit at the University of Birmingham (UK) in accordance with the Home Office guidelines set out in the 1986 Animal Act (UK), adhering to the ARRIVE guidelines and the ARVO Statement for the Use of Animals in Ophthalmic and Vision Research under UK Home Office License number 70/8611.

Adult (8–10 week old) male Sprague Dawley rats (Charles River, Kent, UK) were housed with free access to food and water under a 12 h dark/light cycle. Surgery was performed at the Biomedical Services Unit at the University of Birmingham (UK) in accordance with the Home Office guidelines set out in the 1986 Animal Act (UK) and the ARVO Statement for the Use of Animals in Ophthalmic and Vision Research. Intraocular injections, optical coherence tomography and intraocular pressure measurements were all taken whilst rats were under inhaled anaesthesia using between 2% and 5% isofluorane /98–95% O_2_ (National Veterinary Services, Stoke-on-Trent, UK). The welfare of all rats was monitored closely at all times.

### IOP measurements

IOP measurements were obtained using an iCare Tonolab (ICare, Helsinki, Finland) calibrated for rats. IOP was recorded twice weekly between 9 am and 11 am throughout the 28 day experiment to avoid confounding circadian variabilities in IOP. Immediately after induction of anaesthesia, six rebound measurements were taken with the tonometer from the central cornea and averaged to give a single reading (mmHg). Each graphical data point represents the mean ± SEM of three readings (6 rebounds) taken from each rat sequentially to ensure accurate measurements.

### Optical coherence tomography

RNFL measurements were taken at 28 days using a Spectralis HRA3 confocal scanning laser ophthalmoscope (Heidelberg Engineering, Heidelberg, Germany). Images were obtained from rats whilst under inhalational anaesthesia (as described in the ‘Animals and Surgery’ section above). OCT images were taken of the retina around the optic nerve head and in-built software was used to segment images and quantify the RNFL thickness.

### Tissue preparation for immunohistochemistry

Rats were euthanized by an intraperitoneal injection of sodium pentobarbital (National Veterinary Services) on 28 days and intracardially perfused with 4% paraformaldehyde (PFA; TAAB, Aldermaston, UK) in PBS whilst under terminal anaesthesia. Eyes were removed and immersion fixed in 4% PFA in PBS for 2 h at room temperature and then cryoprotected using increasing concentrations of (10%, 20 and 30% sucrose) for 24 h each at 4 °C. Following this, eyes were embedded in optimal cutting temperature (OCT) embedding medium (Thermo Shandon, Runcorn, UK) in a peel-a-way mould container (Agar Scientific, Essex, UK) and rapidly frozen using crushed dry-ice before storage at −80 °C. Eyes were sectioned (15 µm) in the parasagittal plane through the optic nerve head (to account for RGC variation) at −22 °C using a Bright cryostat microtome (Bright, Huntingdon, UK) and mounted on positively charged glass slides (Superfrost Plus, Fisher Scientific, Loughborough, UK) and left to dry for 2 h at 37 °C and stored at −20 °C.

### Immunohistochemistry

All reagents were purchased from Sigma (Poole, UK) unless otherwise specified. Frozen tissue sections were left for 30 min to equilibrate to room temperature and then hydrated in PBS 3 × 5 min before being immersed in 0.1% Triton X-100 for 20 min to permeabilise the sections. Following a further PBS 3 × 5 min wash, eye sections were isolated with a hydrophobic PAP pen (Vector Laboratories, Peterborough, UK). Non-specific antibody binding sites were blocked using 0.5% BSA, 0.3% Tween-20 and 15% Normal Goat Serum in PBS. Sections were then placed in primary antibody (1/200 rabbit anti-laminin antibody, L9393, Sigma; 1/200 rabbit anti-fibronectin antibody, F3648, Sigma; 1/100 goat anti-Brn3a antibody, SC-31984, Santa Cruz Biotechnology, Dallas, US) and left overnight at 4 °C before being washed 3 × 5 min in PBS and incubated for 1 h at room temperature with secondary antibody (1/400 goat anti-rabbit Alexa Fluor 594, A-11058, or 1/400 donkey anti-goat Alexa Fluor 488, A-11012, both from Thermo Fischer Scientific). Before mounting with Vectashield containing DAPI (Vector Laboratories), sections were washed 3 × 5 min in PBS. Control tissue sections, incubated without primary antibodies but with secondary antibodies, were all negatively stained (not shown).

### Microscopy and analysis

Fluorescently stained sections were analysed by on operator masked to treatment groups using randomised numbers using a Zeiss Axioplan 2 fluorescent microscope (Carl Zeiss Ltd, Oberkochen, Germany) as previously described^5^. Briefly, images for each antibody were captured at the same exposure (250 ms) to assess pixel intensity for ECM levels. The percentage of immunofluorescent pixels above the threshold within the region of interest was measured using the image J software.

### Statistical analysis of in vitro and in vivo data

Values are represented as means and SEM. The data were analysed using GraphPad Prism software and were assessed initially for normality of data. For the TM immunostaining analysis, the non-parametric Wilcoxon Test (two tailed) was used. Two-way ANOVA with Sidak’s multiple comparisons test was used for the comparison of IOP between the groups over a period of time. For the fibronectin, laminin and BRN3a immunohistochemistry, the non-parametric Mann–Whitney test (two tailed) was used, whereas the RNFL layer measurements were normally distributed and so a *T*-test (two tailed) was used. Values were considered statistically significant when *P* values were *P < 0.05, **P < 0.01, ***P < 0.001, and ****P < 0.0001.

### Reporting summary

Further information on research design is available in the Nature Research Reporting Summary linked to this article.

## Supplementary information

Supplementary figures and tables

Reporting Summary Checklist

## Data Availability

The data that support the findings of this study are available from the corresponding author upon reasonable request. The gene expression data have been deposited in the GEO public database repository (accession number: GSE153199).
