# Supplementary material for: ILB® resolves inflammatory scarring and promotes functional tissue repair
Source: NPJ Regen Med. 2021 Jan 7;6:3. doi: 10.1038/s41536-020-00110-2 (PMC7791102; doi:10.1038/s41536-020-00110-2)
Supplement: Supplementary file 1 — Supplementary figures and tables [file 41536_2020_110_MOESM1_ESM.pdf]

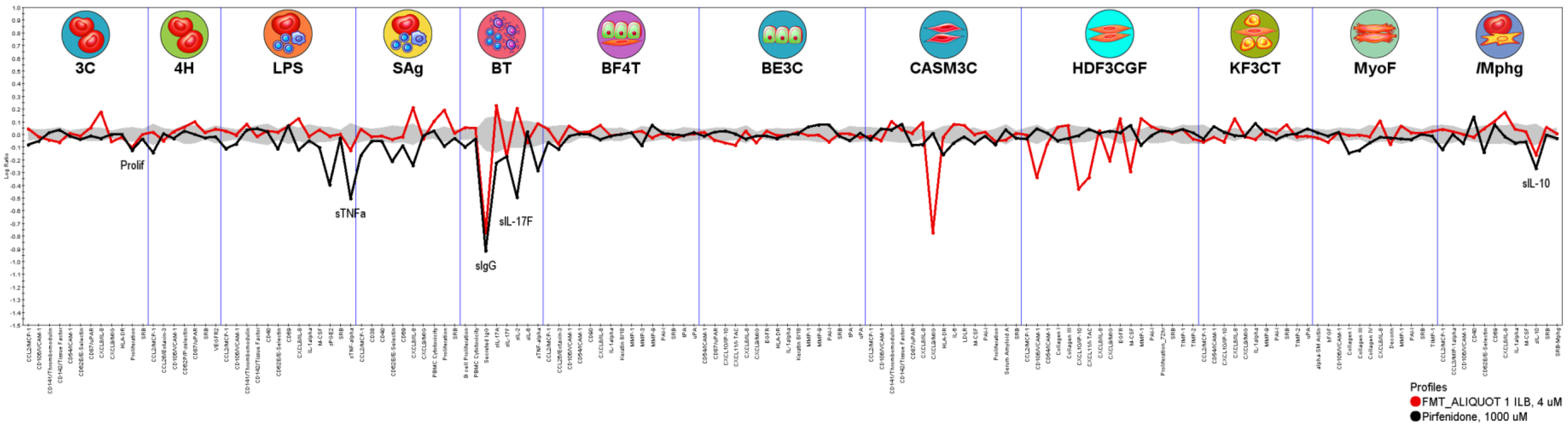

Supplementary Figure 1: Benchmarking ILB® cellular responses against Pirfenidone cellular responses

Log ratio data relating to the cellular and molecular responses of all 12 BioMAP® Diversity Plus human cell systems, 4000nM ILB® (red line) was referenced against 1000µM Pirfenidone (black line) an anti-fibrotic compound approved for the treatment of idiopathic pulmonary fibrosis. The biomarkers assessed in each system are indicated along the x-axis and the grey envelope represents historical vehicle control data at a 95% confidence interval. Biomarkers that are deemed to be significantly altered by ILB® treatment are annotated on the graph (n=minimum of 6).

a

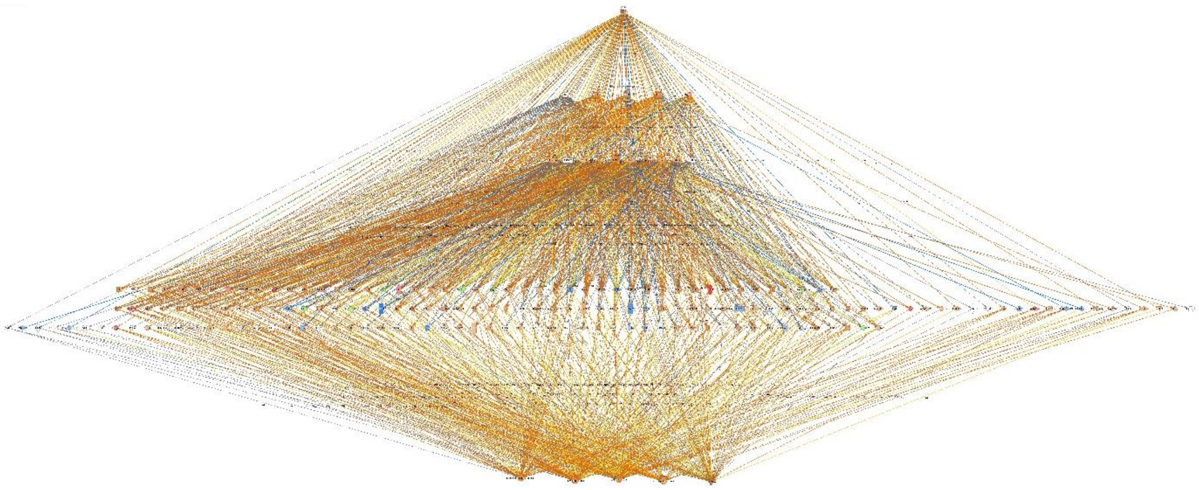

b

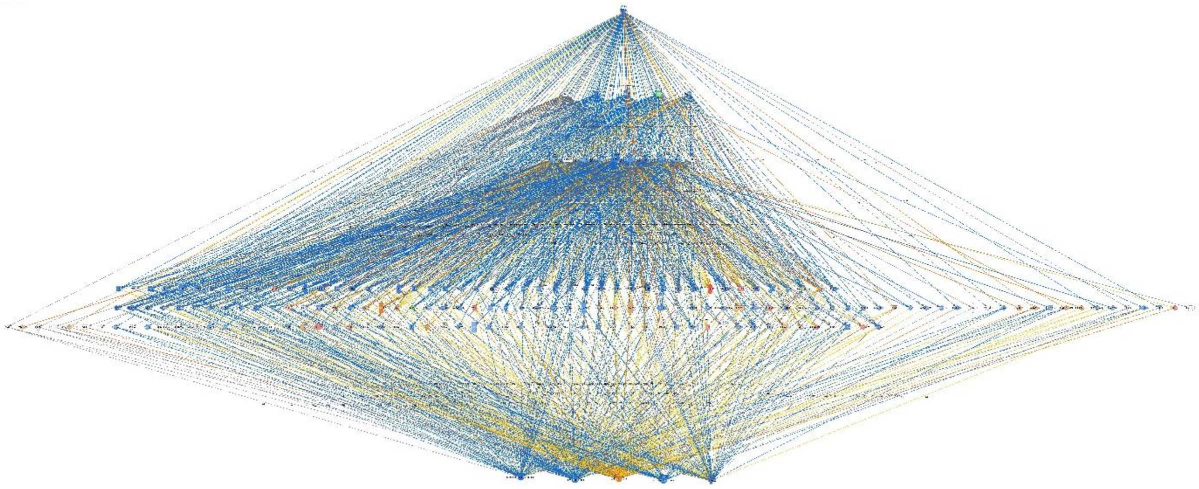

Supplementary Figure 2: The mechanistic molecular network regulated by TGFβ

TGFβ signalling regulates the expression of a large number of molecules. The known molecular targets of TGFβ signalling (derived from the IPA knowledge base) allow the prediction of the downstream functional effects of TGFβ signalling and the construction of a molecular network of molecules that collectively orchestrate fibrogenesis. In this model, initiation of TGFβ signalling elicits a cascade culminating in the activation of immune cells and fibrosis (orange). The gene expression changes seen in the human Schwann cell cultures after 48 hours **(a)** replicate these effects (n=3), while ILB® treatment of the human Schwann cell cultures **(b)** inhibits (blue) these changes (n=3), with the exception of cell movement, despite the fact that ILB® does not directly downregulate TGFβ gene expression itself.

Regulator	Molecules - Mechanistic network, including fibrosis and scarring
TGFβ1	**+A2M**, ACE, ADAM10, ADAM12, **+ADAM17**, ADAM19, ADAMTS1, ADAMTS13, ADAMTS2, ADAMTS4, ADAMTS5, ADAMTS8, **+ADAMTS9**, ADIPOQ, ADORA1, AGT, ALCAM, ANGPT2, ANXA2, APP, AREG, BDNF, BSG, C1QA, C9orf3, CAMP, CCL13, CCL2, CCL23, CCL5, **+CCL7**, CCL8, CD40, CD40LG, CD44, CDH1, CDH11, CDH5, COL4A1, COPS5, CSF3, **+CTGF**, CTTN, CX3CL1, CXCL12, CXCL8, **+DCN**, DDR2, DSG2, EDN1, EDNRB, EFEMP1, EGF, EGFR, ELN, ERAP2, ERBB2, ESR1, F2R, FAS, FASLG, FGF2, FGF7, FN1, FOS, FURIN, **+GDNF**, GNRHR, GPR65, HAVCR1, HBEGF, HIF1A, HMGB1, HSPG2, HTRA1, IFNG, IGFBP3, IGFBP5, IL10, IL13, IL15, IL17A, IL18, IL1A, IL1B, IL1R2, IL1RN, IL32, IL4, **-IL6**, ITGAM, JUN, KDR, KIRREL3, KNG1, LEP, LPAR1, LTA, LTB, LUM, MAOA, MAPK1, MAPK3, MET, MMP1, MMP10, MMP11, MMP12, MMP13, MMP14, MMP15, MMP2, MMP3, MMP7, MMP8, MMP9, NFKBIA, NGF, NGFR, NME1, NOV, NR3C1, OCLN, PAPPA, PCDHGC3, PLAT, PLAU, PLG, **+POSTN**, PPIA, PSEN1, RELA, S100A12, S100A4, SDC1, SDC4, SEMA3A, SERPINA1, SERPINA3, **+SERPINB2**, SERPINE1, **+SERPINE2**, SFTPD, SKIL, SMAD3, SMAD7, SNAP23, SRC, STAT3, TAC1, TERT, TFPI2, TGFA, TGFB1, **+TGIF1**, TIMP1, TIMP2, TIMP3, TIMP4, **+TNC**, TNF, TNFSF11, **+VCAM1**, VEGFA, VHL

Supplementary Table 1: The effect of ILB® treatment on the expression of genes of the TGFβ regulated mechanistic network that controls fibrosis and scarring (data derived from Agilent gene array analysis)

The gene expression of many molecules in the TGFβ1 mechanistic network showed no change, although the expression of those genes marked as **+Bold** were upregulated and those marked as **-Bold** were down regulated, relative to the control.

Ingenuity canonical pathways	Pathways affected by culture	Pathways affected by ILB®	Pathways affected by TGFβ1	Pathways affected by other scar regulators	Pathways affected by heparin
Inhibition of Matrix Metalloproteases	YES		YES	YES	YES
Role of Macrophages, Fibroblasts and Endothelial Cells in Rheumatoid Arthritis	YES	YES	YES	YES	YES
Coagulation System	YES		YES	YES	YES
Interferon Signaling	YES		YES	YES	YES
Atherosclerosis Signaling	YES		YES	YES	YES
PPARα/RXRα Activation	YES		YES	YES	YES
Leukocyte Extravasation Signaling	YES	YES	YES	YES	YES
IL-3 Signaling	YES		YES	YES	YES
Role of PKR in Interferon Induction and Antiviral Response	YES		YES	YES	YES
Dendritic Cell Maturation	YES		YES	YES	YES
IL-9 Signaling	YES		YES	YES	YES
IL-1 Signaling	YES		YES	YES	YES
IL-15 Production	YES	YES	YES	YES	
IL-12 Signaling and Production in Macrophages	YES		YES	YES	YES
Granulocyte Adhesion and Diapedesis	YES		YES	YES	YES
VEGF Signaling	YES		YES	YES	YES
eNOS Signaling	YES		YES	YES	YES
JAK/Stat Signaling	YES		YES	YES	YES
B Cell Development	YES			YES	YES
IL-17 Signaling	YES		YES	YES	YES
Role of IL-17F in Allergic Inflammatory Airway Diseases		YES	YES	YES	YES
IL-6 Signaling		YES	YES	YES	YES
Neuregulin Signaling		YES	YES	YES	YES
IL-17A Signaling in Fibroblasts		YES	YES	YES	YES
ERK/MAPK Signaling		YES	YES	YES	YES
TGF-β Signaling		YES	YES	YES	YES
Wnt/β-catenin Signaling		YES	YES	YES	YES
Acute Phase Response Signaling		YES	YES	YES	YES
IL-10 Signaling		YES	YES	YES	YES
ErbB Signaling		YES	YES	YES	YES
IL-15 Signaling		YES	YES	YES	YES
Regulation of the Epithelial-Mesenchymal Transition Pathway		YES	YES	YES	YES

Supplementary Table 2: Canonical pathways significantly affected in human Schwann cell cultures (derived from the Agilent gene expression data obtained from cultures without and with ILB® treatment) compared to the canonical pathways known to be affected by TGFβ and other scar regulators, including heparin
