# Supplementary material for: ILB® resolves inflammatory scarring and promotes functional tissue repair
Source: NPJ Regen Med. 2021 Jan 7;6:3. doi: 10.1038/s41536-020-00110-2 (PMC7791102; doi:10.1038/s41536-020-00110-2)
Supplement: Supplementary file 2 — Reporting Summary Checklist [file 41536_2020_110_MOESM2_ESM.pdf]

nature research

Corresponding author(s): Professor Ann Logan
ORCID ID: 0000-0003-3215-5042
Axolotl Consulting Ltd, Droitwich, Worcestershire, WR9 0JS, UK.

Last updated by author(s): Jun 18, 2020

Reporting Summary

Nature Research wishes to improve the reproducibility of the work that we publish. This form provides structure for consistency and transparency in reporting. For further information on Nature Research policies, see our Editorial Policies and the Editorial Policy Checklist.

Statistics

For all statistical analyses, confirm that the following items are present in the figure legend, table legend, main text, or Methods section.

n/a	Confirmed	
☐	☒	The exact sample size (*n*) for each experimental group/condition, given as a discrete number and unit of measurement
☐	☒	A statement on whether measurements were taken from distinct samples or whether the same sample was measured repeatedly
☐	☒	The statistical test(s) used AND whether they are one- or two-sided<br>*Only common tests should be described solely by name; describe more complex techniques in the Methods section.*
☒	☐	A description of all covariates tested
☐	☒	A description of any assumptions or corrections, such as tests of normality and adjustment for multiple comparisons
☐	☒	A full description of the statistical parameters including central tendency (e.g. means) or other basic estimates (e.g. regression coefficient) AND variation (e.g. standard deviation) or associated estimates of uncertainty (e.g. confidence intervals)
☐	☒	For null hypothesis testing, the test statistic (e.g. *F*, *t*, *r*) with confidence intervals, effect sizes, degrees of freedom and *P* value noted<br>*Give P values as exact values whenever suitable.*
☒	☐	For Bayesian analysis, information on the choice of priors and Markov chain Monte Carlo settings
☒	☐	For hierarchical and complex designs, identification of the appropriate level for tests and full reporting of outcomes
☒	☐	Estimates of effect sizes (e.g. Cohen's *d*, Pearson's *r*), indicating how they were calculated

Our web collection on statistics for biologists contains articles on many of the points above.

Software and code

Policy information about availability of computer code

Data collection	No software was used for data collection.
Data analysis	ImageJ; GraphPad; MetaboAnalyst; Yokogawa image analysis software.

For manuscripts utilizing custom algorithms or software that are central to the research but not yet described in published literature, software must be made available to editors and reviewers. We strongly encourage code deposition in a community repository (e.g. GitHub). See the Nature Research guidelines for submitting code & software for further information.

Data

Policy information about availability of data

All manuscripts must include a data availability statement. This statement should provide the following information, where applicable:

- Accession codes, unique identifiers, or web links for publicly available datasets
- A list of figures that have associated raw data
- A description of any restrictions on data availability

The data that support the findings of this study are available from the corresponding author upon reasonable request. The gene expression data has been deposited in the GEO public database repository (accession number: GSE153199, https://www.ncbi.nlm.nih.gov/geo/query/acc.cgi?acc=GSE153199).

Field-specific reporting

Please select the one below that is the best fit for your research. If you are not sure, read the appropriate sections before making your selection.

☒ Life sciences ☐ Behavioural & social sciences ☐ Ecological, evolutionary & environmental sciences

For a reference copy of the document with all sections, see nature.com/documents/nr-reporting-summary-flat.pdf

Life sciences study design

All studies must disclose on these points even when the disclosure is negative.

Sample size	The sample sizes were based on power calculations using previous data sets to a power of 0.8 with significance at p<0.05
Data exclusions	Data sets were excluded in the in vivo and gene expression studies and a statement within the manuscript has been included where applicable.
Replication	Replicates for experiments were successful and are reflected within the results.
Randomization	For in vivo studies all rats were randomly allocated into treatment groups using computer randomisation.
Blinding	Images were assigned randomized file names to ensure masking of treatment groups for the assessor.

Reporting for specific materials, systems and methods

We require information from authors about some types of materials, experimental systems and methods used in many studies. Here, indicate whether each material, system or method listed is relevant to your study. If you are not sure if a list item applies to your research, read the appropriate section before selecting a response.

Materials & experimental systems

n/a	Involved in the study	
☐	☒	Antibodies
☐	☒	Eukaryotic cell lines
☒	☐	Palaeontology and archaeology
☐	☒	Animals and other organisms
☒	☐	Human research participants
☒	☐	Clinical data
☒	☐	Dual use research of concern

Methods

n/a	Involved in the study	
☒	☐	ChIP-seq
☒	☐	Flow cytometry
☒	☐	MRI-based neuroimaging

Antibodies

Antibodies used	Rabbit anti-laminin in rabbit, Sigma, L393 Batch 015M4881V; Rabbit anti-fibronectin, Sigma, F3648, batch 125M4835V; Goat anti-BRN3A, Santa Cruz SC-31984; Mouse anti-human Fibronectin Alexa Fluor® 488, Thermo Fisher Scientific, 53-9869-82; Goat anti-rabbit Alexa Fluor 594, A-11058, Thermo Fisher Scientific; Donkey anti-goat Alexa Fluor 488, Thermo Fisher Scientific
Validation	All antibodies have been previously optimized in our laboratory and are used routinely (e.g. PMID: 26066743; PMID: 24384090). Websites (listed below) have many citations for each of the antibodies listed. Negative (no primary controls we used for all experiments and is stated within the methods of this manuscript).<br><br>Rabbit anti-laminin antibody, Sigma, L393; https://www.sigmaaldrich.com/catalog/product/sigma/l9393?lang=en&region=GB<br>Specificity of the anti-laminin antibody is determined by indirect immunofluorescent labeling of formalin-fixed, paraffin-embedded human or animal tissue sections, and by dot blot immunoassay. By indirect immunofluorescence the antibody demonstrates specific basement membrane staining of enzymatically unmasked human and animal tissue. In the dot blot immunoassay the rabbit anti-laminin antibody reacts with laminin but not with fibronectin, vitronectin, collagen IV, or chondroitin sulfate types A, B, and C. The affinity isolated antibody to laminin will react with laminin of human, mammal, avian, reptilian, and amphibian sources.<br>The antibody shows no cross-reaction with collagen type IV, fibronectin or chondroitin sulfate types A, B, and C using a dot blot immunoassay. Rabbit Anti-Laminin antibody may be used in immunohistochemistry for marking blood vessel walls in different species, classification of various disease processes involving basement membranes, identification of the origin of human tumors and their classification, and for distinguishing between non-invasive and invasive lesions. The antibody may be used to monitor levels of laminin in biological fluids and for experimental production of basement membrane lesions in vivo.<br>A working dilution of at least 1:1,000 was determined by a dot blot immunoassay using laminin at 50 ng per dot.<br>A working dilution of at least 1:25 was determined by indirect immunohistology using formalin-fixed, paraffin-embedded human and animal tissues.<br><br>Rabbit anti-fibronectin antibody; Sigma F3648 https://www.sigmaaldrich.com/catalog/product/sigma/f3648?lang=en&region=GB

Specificity The anti-fibronctin antibody shows no cross-reaction with laminin, vitronectin, collagen type IV, or chondroitin sulfate types A, B and C using a dot blot immunoassay.
The asntiserum is produced in rabbit using purified human fibronectin as the immunogen. Affinity isolated antibody is obtained from antiserum by immunospecific purification which removes essentially all rabbit serum proteins, including immunoglobulins, which do not specifically bind to human fibronectin. The antiserum is determined to be immunospecific for human fibronectin by immunofluorescent labeling of human fibroblast cell cultures, ELISA and immunoblotting. In immunoblotting, a specific band of fibronectin at 220 kDa is observed (another band at 94 kDa may be also be present) using human fibronectin. When used in immunoelectrophoresis, the antibody shows 1-2 arcs of precipitation versus normal human plasma. This product may be used for immunohistochemical localization of fibronectin in normal, inflamed and neoplastic tissues, for detection of fibronectin on cultured cells and structure and function studies of fibronectins in human and animal body fluids, tissues and cells. Affinity isolated antibody to human fibronectin can be used for immunofluorescent and immunoperoxidase staining of cultured cells, frozen sections and formalin fixed, paraffin-embedded tissues. Other fixatives, e.g. methacarn and ethanol, may also be used.

Goat anti-Brn3a antibody; SC-31984, Santa Cruz, http://datasheets.scbt.com/sc-31984.pdf
The anti-Brn-3a (C-20) antibody is recommended for detection of Brn-3a of mouse, rat and human origin by Western Blotting (starting dilution 1:200, dilution range 1:100-1:1000), immunoprecipitation [1-2 µg per 100-500 µg of total protein (1 ml of cell lysate)], immunofluorescence (starting dilution 1:50, dilution range 1:50-1:500) and solid phase ELISA (starting dilution 1:30, dilution range 1:30-1:3000).
Brn-3a (C-20) is also recommended for detection of Brn-3a in additional species, including canine.
Suitable for use as control antibody for Brn-3a siRNA (h): sc-29839, Brn-3a siRNA (m): sc-29840, Brn-3a shRNA Plasmid (h): sc-29839-SH, Brn-3a shRNA Plasmid (m): sc-29840-SH, Brn-3a shRNA (h) Lentiviral Particles: sc-29839-V
and Brn-3a shRNA (m) Lentiviral Particles: sc-29840-V.
Brn-3a (C-20) X TransCruz antibody is recommended for Gel Supershift and ChIP applications.
Molecular Weight (predicted) of Brn-3a: 43 kDa.
Molecular Weight (observed) of Brn-3a: 47 kDa.
Positive Controls: Brn-3a (h): 293T Lysate: sc-128117.

Mouse anti-human Fibronectin Alexa Fluor® 488; 53-9869-82, Thermo Fisher Scientific
The FN-3 monoclonal antibody recognizes human fibronectin, a component of the extracellular matrix. This high molecular weight protein (almost 400 kDa) exists as an extracellular and a soluble plasma form. Fibronectin functions in cell adhesion and migration via integrins present on the interacting cell. Fibronectin also associates with collagen, actin and fibrins. In malignancies, fibronectin protein levels can be altered. In lung carninomas, expression is decreased. The FN-3 antibody recognizes a determinant on human cellular but not plasma fibronectin; this recognition is not lost upon trypsin treatment. Furthermore, FN-3 antibody has been shown to crossreact to bovine fibronectin.
Applications Tested: This FN-3 antibody has been tested by immunohistochemistry on FFPE human placenta (with IHC Antigen Retrieval Solution - Low pH (cat. 00-4955)) and can be used at less than or equal to 10 µg/mL. Is is recommended that the antibody be carefully titrated for optimal performance in the assay of interest.
Excitation: 488 nm; Emission: 519 nm; Laser: Blue Laser.
Filtration: 0.2 µm post-manufacturing filtered.

Goat anti-rabbit Alexa Fluor 594; A-11058, Thermo Fisher Scientific
To minimize cross-reactivity, these goat anti-rabbit IgG whole antibodies have been cross-adsorbed against human IgG, human serum, mouse IgG, mouse serum, and bovine serum. Cross-adsorption or pre-adsorption is a purification step to increase specificity of the antibody resulting in higher sensitivity and less background staining. The secondary antibody solution is passed through a column matrix containing immobilized serum proteins from potentially cross-reactive species. Only the nonspecific-binding secondary antibodies are captured in the column, and the highly specific secondaries flow through. The benefits of this extra step are apparent in multiplexing/multicolor-staining experiments (e.g., flow cytometry) where there is potential cross-reactivity with other primary antibodies or in tissue/cell fluorescent staining experiments where there are may be the presence of endogenous immunoglobulins.
The anti-Rabbit secondary antibodies are affinity-purified antibodies with well-characterized specificity for rabbit immunoglobulins and are useful in the detection, sorting or purification of its specified target. Secondary antibodies offer increased versatility enabling users to use many detection systems (e.g. HRP, AP, fluorescence).

Donkey anti-goat Alexa Fluor 488; A-11012, Thermo Fisher Scientific
To minimize cross-reactivity, these donkey anti-goat IgG whole antibodies have been cross-adsorbed against rabbit, rat, mouse, and human IgG. Cross-adsorption or pre-adsorption is a purification step to increase specificity of the antibody resulting in higher sensitivity and less background staining. The secondary antibody solution is passed through a column matrix containing immobilized serum proteins from potentially cross-reactive species. Only the nonspecific-binding secondary antibodies are captured in the column, and the highly specific secondaries flow through.

Eukaryotic cell lines

Policy information about cell lines

Cell line source(s)	sNF96.2(ATCC® CRL-2884™), ATTC, Teddington, UK; TMC-6590, ScienCell Research Laboratories, Carlsbad, CA.
Authentication	None of the cell lines were authenticated
Mycoplasma contamination	CRL-2884™ and TMC-6590 cells were tested for mycoplasma by the supplier and sent to us mycoplasma free.
Commonly misidentified lines (See ICLAC register)	n/a

Animals and other organisms

Policy information about studies involving animals; ARRIVE guidelines recommended for reporting animal research

Laboratory animals	Adult (8-10 week old) male Sprague Dawley rats (Charles River, Kent, UK)
Wild animals	The study did not involve wild animals
Field-collected samples	The study did not involve field collected samples
Ethics oversight	All ethical approvals have been detailed in the manuscript. Surgery was performed at the Biomedical Services Unit at the University of Birmingham (UK) in accordance with the Home Office guidelines set out in the 1986 Animal Act (UK) and the ARVO Statement for the Use of Animals in Ophthalmic and Vision Research.

Note that full information on the approval of the study protocol must also be provided in the manuscript.
